# Ordinal Prediction Model of 90-Day Modified Rankin Scale in Ischemic Stroke

**DOI:** 10.3389/fneur.2021.727171

**Published:** 2021-10-22

**Authors:** Michelle Y. Zhang, Michael Mlynash, Kristin L. Sainani, Gregory W. Albers, Maarten G. Lansberg

**Affiliations:** ^1^Stanford University School of Medicine, Stanford, CA, United States; ^2^Department of Neurology and Neurological Sciences and the Stanford Stroke Center, Stanford University Medical Center, Stanford, CA, United States; ^3^Department of Epidemiology and Population Health, Stanford University, Stanford, CA, United States

**Keywords:** modified rankin scale, neurology, ischemic stroke, outcome, ordinal regression

## Abstract

**Background and Purpose:** Prediction models for functional outcomes after ischemic stroke are useful for statistical analyses in clinical trials and guiding patient expectations. While there are models predicting dichotomous functional outcomes after ischemic stroke, there are no models that predict ordinal mRS outcomes. We aimed to create a model that predicts, at the time of hospital discharge, a patient's modified Rankin Scale (mRS) score on day 90 after ischemic stroke.

**Methods:** We used data from three multi-center prospective studies: CRISP, DEFUSE 2, and DEFUSE 3 to derive and validate an ordinal logistic regression model that predicts the 90-day mRS score based on variables available during the stroke hospitalization. Forward selection was used to retain independent significant variables in the multivariable model.

**Results:** The prediction model was derived using data on 297 stroke patients from the CRISP and DEFUSE 2 studies. National Institutes of Health Stroke Scale (NIHSS) at discharge and age were retained as significant (*p* < 0.001) independent predictors of the 90-day mRS score. When applied to the external validation set (DEFUSE 3, *n* = 160), the model accurately predicted the 90-day mRS score within one point for 78% of the patients in the validation cohort.

**Conclusions:** A simple model using age and NIHSS score at time of discharge can predict 90-day mRS scores in patients with ischemic stroke. This model can be useful for prognostication in routine clinical care and to impute missing data in clinical trials.

## Introduction

Prediction models of functional outcome after ischemic stroke can aid clinical decision making for providers, patients, and families by guiding rehabilitation goals, discharge planning, and patient expectations (1–3). They can also be useful for imputing missing data in clinical trials. These models stroke have generally focused on predicting a dichotomization of the modified Rankin Scale (mRS) such as functional independence (mRS 0–2) vs. functional dependency or death (mRS 3–6), or alive (mRS 0–5) vs. dead (mRS 6) (4–6). While these dichotomizations are meaningful, a model that could predict outcome across the entire spectrum of the mRS would be more informative. For example, for patients who have less severe strokes, a model predicting mortality may be less useful than a model that predicts the exact score on the mRS (7). Such a model could also be used to impute missing data in clinical trials when patients are lost to follow-up or when outcome data is not yet available.

### Aims

To address this need, we aimed to develop an ordinal logistic regression model that predicts the 90-day mRS score based on variables available at the time of hospital discharge.

## Methods

The data that support the findings of this study are available from the corresponding author upon reasonable request.

### Study Patients

This study used de-identified patient data from three prior studies: CRISP, DEFUSE 2, and DEFUSE 3 (8–10). CRISP and DEFUSE 2 were multi-center prospective cohort studies and DEFUSE 3 was a prospective randomized open-label multicenter trial of endovascular therapy. Patients were older than 18, and only DEFUSE3 had an upper age limit of 90 years. Other inclusion criteria included diagnosis of ischemic stroke, eligibility for endovascular therapy, baseline NIHSS ≥ 5 (CRISP, DEFUSE 2) or ≥6 (DEFUSE 3). All studies received approval from local institutional review boards, and patients or their proxies provided written informed consent.

### Variables

The dependent variable was the mRS score obtained 90 days after the index event. For patients with missing 90-day mRS scores, 30-day mRS scores were carried forward. Patients missing both outcome measures were excluded from the study. Patients who died during the initial hospitalization were also excluded.

Predictor variables included baseline characteristics [sex, age, history of atrial fibrillation, history of diabetes, history of hypertension, history of stroke or transient ischemic stroke (TIA)], imaging measures (24-h follow-up infarct volume, hemorrhagic transformation per the European Cooperative Acute Stroke Study criteria), and clinical measures (NIHSS score at discharge or day 5 of hospitalization, whichever occurred earlier).

### Model Derivation

The CRISP and DEFUSE 2 datasets were used as the derivation set. The ordinal outcome measure was the mRS score at 90 days. We used a proportional odds model, which estimates intercepts for each level, but assumes a common coefficient across ordered response categories. Validity of the proportional odds assumption was verified by trending univariate odds ratios for each cutoff and plotting partial residuals. The derivation dataset was “upsampled” to account for the relatively small number of participants with an mRS score of five at day 90. The upsampling method augments the minority class by sampling random observations from this class with replacement (i.e., bootstrapping). We implemented upsampling using the upsample function in the package *R*-splitters. After upsampling, variables that were associated with outcome at a *p* < 0.2 in univariate analysis were entered in a multivariable model and were retained if they reduced the AIC by seven or more points.

### Model Validation

The model was internally and externally validated to assess model performance on unseen data, thereby mitigating possible overfitting of the model. The model was internally validated within the derivation set using five-fold cross-validation. Univariate screening and forward selection were repeated to derive a model for each fold. The model was externally validated using the DEFUSE 3 dataset.

Measures used to evaluate for model performance included *R*^2^ (coefficient of determination), mean absolute error (MAE), and the percentage of predicted outcomes that fell within one point of the observed outcomes. As an additional measure of model performance, we ran the primary efficacy analysis of the DEFUSE 3 trial based on imputed 90-day mRS scores and compared it to the same analysis using observed 90-day mRS scores.

### Statistical Analyses

Proportions were compared using Fisher's exact test, and distributions of continuous and ordinal variables were compared using the *t*-test or Wilcoxon rank-sum test. We report two-sided results and used a *p* < 0.05 as a threshold for statistical significance.

All statistical analyses were performed using R software (version 3.6.2) and SAS software (version 9.4).

## Results

There were 201 patients enrolled in the CRISP study, 130 in DEFUSE 2, and 182 in DEFUSE 3. In the derivation set (CRISP and DEFUSE 2), we excluded two patients with missing 30 and 90-day mRS outcome data, and 32 patients who died during their initial hospitalization. In the validation set (DEFUSE 3), we excluded 22 patients who died during their initial hospitalization. (Supplementary Figure I). The patient characteristics are presented in Table 1. Age (65.6 vs. 68.9, *p* = 0.02), prevalence of hypertension (66.7 vs. 78.8%, *p* = 0.01), hemorrhagic transformation score (*p* = 0.01), NIHSS score at discharge (6 vs. 8, *p* = 0.03), and infarct volume at early follow up (26.2 vs. 37.7 ml, *p* < 0.001) were different between the derivation and validation groups.

**Table 1:** Characteristics of patients included in the derivation and validation set.

	**Derivation set (*n* = 297)**	**Validation set (*n* = 160)**
Age,^†^ mean (SD)	65.6 (15.3)	68.9 (13.2)
Sex (female), *n* (%)	143 (48.1%)	82 (51.2%)
Hypertension,^‡^ *n* (%)	198 (66.7%)	126 (78.8%)
Diabetes mellitus, *n* (%)	64 (21.5%)	43 (26.9%)
History of stroke or TIA, ^§^*n* (%)	43 (14.5%)	20 (12.5%)
History of atrial fibrillation, *n* (%)	97 (32.7%)	53 (33.1%)
Hemorrhagic transformation^||^
None, *n* (%)	121 (40.7%)	83 (51.9%)
Hemorrhage infarction type 1 (HI1), *n* (%)	58 (19.5%)	34 (21.2%)
Hemorrhage infarction type 2 (HI2), *n* (%)	51 (17.2%)	27 (16.9%)
Parenchymal hematoma type 1 (PH1), *n* (%)	43 (14.5%)	8 (5.0%)
Parenchymal hematoma type 2 (PH2), *n* (%)	23 (7.7%)	8 (5.0%)
NIHSS score at discharge, ^¶^ median (IQR)	6 (2–14)	8 (3–16)
Infarct volume at early follow up, ^**^ median (IQR)	26.2 (10.6–67.5)	37.7 (22.5–89.9)
Premorbid mRS, median (IQR)	0 (0–0)	0 (0–0)

† *p = 0.02;*

‡ *p = 0.01;*

§ *missing value: derivation set (n = 1);*

|| *p = 0.01, graded per European cooperative acute stroke study criteria, missing value: derivation set (n = 1);*

¶ *p = 0.03;*

** *missing values: derivation set (n = 44), p < 0.001*.

*Wilcoxon rank-sum test for age; Fisher's exact test for sex, hypertension, history of stroke or TIA, history of smoking, and history of atrial fibrillation*.

In the derivation set, predictors that were associated (*p* < 0.2) with the 90-day mRS score in univariate analyses, included age (*p* < 0.001), hypertension (*p* < 0.001), diabetes mellitus (*p* < 0.001), history of stroke (*p* = 0.12), hemorrhagic transformation (*p* < 0.001), NIHSS score at discharge (*p* < 0.001), infarct volume at early follow-up (*p* < 0.001). After forward selection, age and the NIHSS score at discharge remained as independent predictors of the 90-day mRS score in the multivariable ordinal regression model (*p* < 0.001 for each, Table 2).

**Table 2:** Ordinal logistic regression predicting 90-day mRS derived from the full derivation set (*n* = 297).

	**Coefficient**	**95% Confidence interval**
mRS 0|1	2.94	2.10–3.78
mRS 1|2	4.35	3.47–5.23
mRS 2|3	5.62	4.70–6.54
mRS 3|4	7.06	6.04–8.08
mRS 4|5	8.55	7.43–9.67
mRS 5|6	10.14	8.77–11.51
Age	0.05	0.04–0.06
NIHSS score at discharge	0.31	0.27–0.34

**Figure 1:**
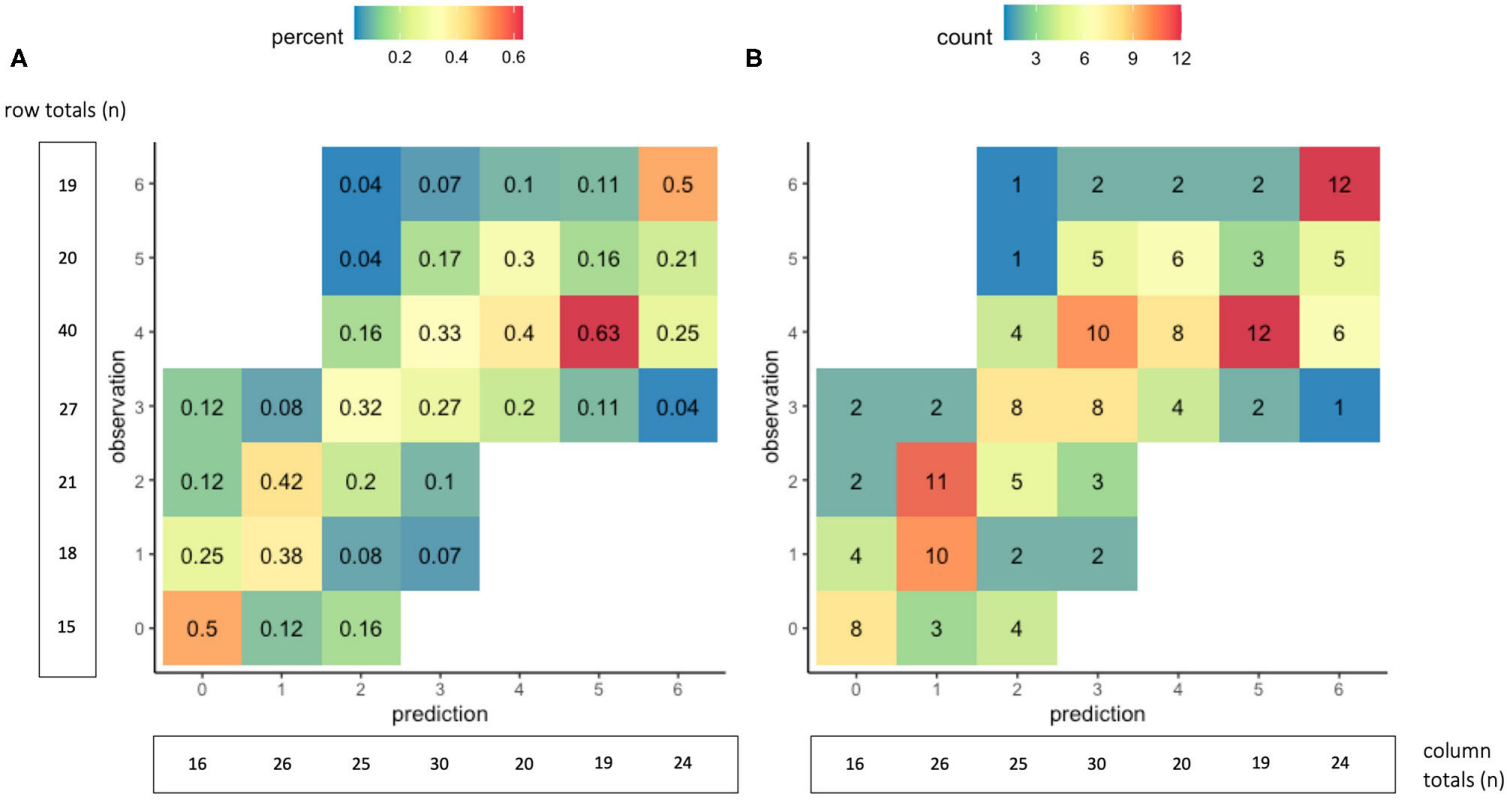
Accuracy of the 90-day mRS prediction model in external validation. The graphs show cross-tabulations between the observed and predicted values of the 90-day mRS for the 160 patients in the external validation group. Values in graph **(A)** show proportions, where the columns represent the distribution of observed outcomes for a given predicted outcome. For example, of the 16 patients with a predicted mRS score of 0, the observed 90-day mRS score was 0 in 50%, one in 25%, two in 12.5%, and three in 12.5%. Values in graph **(B)** show counts of the observed and predicted mRS scores at 90 days.

With five-fold internal cross-validation, age and NIHSS were retained in each model and no other variable was retained in a majority of models. The mean *R*^2^ of the five validation models was 0.60, the average mean absolute error was 0.88 (95% CI 0.82–0.94), and a mean of 80% of predictions were within one of the observed value. Additional results of the five-fold internal cross-validation are shown in Table 3 and Supplementary Figure II. In external validation, using the DEFUSE 3 dataset, the model had an *R*^2^ of 0.60, and a mean absolute error of 0.94 (95% CI 0.80–1.07). The model predicted 34% of the 90-day mRS scores correctly, 78% of the scores within one point of the observed value, 96% within two points of the observed value, and 99% within three points of the observed value. Additional performance metrics of the model in external validation are presented in Table 3 and illustrated in Figure 1. There was no significant difference in the model's performance when validated on the subset of DEFUSE 3 patients who were treated with endovascular therapy and those who were treated with medical management alone (MAE 0.85 vs. 1.04, *p* = 0.15, Supplementary Figure III). We also compared the model in the subset of DEFUSE 3 patients with more severe disability at discharge or day 5 (75% quantile: NIHSS ≥ 16), and there was no significant difference in the models' performance (MAE 0.90 vs. 0.95, *p* = 0.75, Supplementary Figure IV).

**Table 3:** Performance metrics of the 90-day mRS prediction model in internal and external validation.

	***R* ^**2**^**	**MAE (95% CI)**	**% correct**	**% correct within 1 point**	**% correct within 2 points**	**% correct within 3 points**
Internal cross-validation^†^	0.60	0.88 (0.82–0.94)	37	80	96	99
External validation (DEFUSE 3 trial data)
Combined control and EVT arms	0.60	0.94 (0.80–1.07)	34	78	96	99
Control arm only	0.50	1.04 (0.84–1.24)	29	74	95	99
EVT arm only	0.62	0.85 (0.67–1.02)	38	81	96	100

*R^2^, correlation coefficient*.

*MAE, mean absolute error*.

*EVT, endovascular therapy*.

† *values represent means and 95% confidence interval of the values from the five cross-validation iterations*.

The primary analysis of the DEFUSE 3 trial, a comparison of the distribution of 90-day mRS scores between patients in the endovascular treatment and control groups, was rerun with 90-day mRS scores predicted by the final model. The odds ratio for benefit from endovascular therapy using predicted mRS scores was 5.01 (95% CI 2.77–9.05), which is similar to the results of the same analysis performed using observed mRS scores (OR 3.45, 95% 1.94–6.13).

## Discussion

A prediction model based on two easily obtainable clinical variables, age and NIHSS score assessed at the time of hospital discharge, had moderately high accuracy for predicting a patient's 90-day modified Rankin Scale score. It accurately predicted the 90-day mRS within one point in ~ 80% of patients. The model's independent variables — age and NIHSS score — are corroborated by prior studies that have demonstrated that these factors are correlated with functional outcome after ischemic stroke (11–14). This model can be used for prognostication in clinical practice and for imputation of 90-day outcome data in clinical trials.

Several prior studies have focused on predicting survival or other dichotomized functional outcomes after ischemic stroke (5, 6). A recent meta-analysis by Fahey et al. identified over 60 models for outcomes following ischemic stroke (4). Variables such as sex, age, disease characteristics, and comorbidities were the best predictors for mortality and functional outcomes after ischemic stroke. The meta-analysis noted that a limitation of current models is that few models are externally validated (4). In addition, because the current models were derived with dichotomous outcome data, none predict a patient's exact score on the mRS at long-term follow-up. In contrast, our externally validated ordinal logistic regression model predicts a patient's 90-day mRS score with moderately high accuracy.

One of the relative advantages of an ordinal regression is that there is less information loss as compared to dichotomous outcome models. Multiple studies have shown that ordinal analyses increase statistical power and efficiency, and suggest that further clinical research could benefit from increased utilization of ordinal analyses where relevant (15–17). Because of this, most recent acute stroke studies use an ordinal model for their primary outcome analysis. Unlike dichotomous prediction models, our model could be used to impute missing outcomes if 30- and 90-day mRS data are missing. Moreover, an ordinal prognostic model could help optimize future randomized trials of stroke rehabilitation interventions, by excluding patients who are likely to be non-responders because they have either a high chance of spontaneous recovery or a high chance of mortality (18). In addition, our prediction model could be used in clinical practice as a tool to assist with the assessment of a patient's prognosis. This could improve stroke rehabilitation by personalizing rehabilitation plans, reducing variation in therapy, and increasing equity of services. A recent review of prediction tools for stroke rehabilitation found that models were most helpful if they were available at the time of rehabilitation or discharge planning and predicted functional status beyond binary outcomes. The authors were concerned that a general prognosis of good or poor is not sufficiently detailed to be useful (6). Thus, 90-day ordinal predictions could be a helpful reference in discussions with the patient and family and provide additional context when discussing rehabilitation and future residence destination (19). Even though our model is only able to predict well within one point, we believe this presents an advantage over dichotomous models which predict a range without identifying the most likely outcome within that range.

Some researchers have cited concern about the use of ordinal regression models and the need to test for proportionality of the odds. To address this, we confirmed proportionality by visual inspection of the univariate odds ratios and partial residuals. In addition, we empirically demonstrated the model's performance by validating its accuracy in an external dataset.

There are limitations to our study. First, our dataset is limited to patients from three endovascular stroke therapy studies and is therefore not representative of all patients with ischemic strokes. Specifically, the model may not apply to patients with mild strokes or strokes in the posterior circulation who were not eligible for the trials. The model may also not apply well to patients with pre-existing severe disability who were excluded from the studies. The model did perform similarly in patients who underwent endovascular therapy and those who did not, likely because the effect of endovascular therapy is captured in the NIHSS score at discharge, which is one of the prediction variables in the model (20, 21) Future studies validating this model in a larger cohort could provide additional information about the generalizability of this model. Second, while the simplicity of our model, which only includes two predictor variables, is a relative strength, a model derived from an even larger dataset could identify additional independent variables that might further improve the model's performance.

## Conclusions

In summary, our internally and externally validated model predicts the ordinal mRS score at 90 days after ischemic stroke with moderately good accuracy and could be used for prognostication in clinical practice and to impute missing data in clinical trials.

## Data Availability Statement

The raw data supporting the conclusions of this article will be made available by the authors, without undue reservation.

## Ethics Statement

The studies involving human participants were reviewed and approved by the Stanford IRB (DEFUSE 2 and CRISP) and the University of Cincinnati IRB serving as the Central IRB for StrokeNet (DEFUSE 3). The patients/participants provided their written informed consent to participate in this study.

## Author Contributions

ML and MM organized the database. MZ wrote the first draft of the manuscript. All authors contributed to conception and design of the study, statistical analysis, manuscript revision, read, and approved the submitted version.

## Funding

The study was funded by grants from the National Institute for Neurological Disorders and Stroke (principal investigators, ML and GA; 1TR01ActNS075209Project01A1Year, 5TR01ActNS075209Project02Year, 5TR01ActNS075209Project03Year, 5TR01ActNS075209Project04Year, 5TR01ActNS075209Project05Year, 2TR01ActNS075209Project06Year, 5TR01ActNS075209Project07Year, 5TR01ActNS075209Project08Year, 1TK23ActNS051372Project01A1Year, 5TK23ActNS051372Project02Year, 5TK23ActNS051372Project03Year, 5TK23ActNS051372Project04Year, 3TK23ActNS051372Project04S1Year, 5TK23ActNS051372Project05Year, 1TU10ActNS086487Project01Year, 5TU10ActNS086487Project02Year, 5TU10ActNS086487Project03Year, 4TU10ActNS086487Project04Year, 5TU10ActNS086487Project05Year, 1TU01ActNS092076Project01A1Year, 5TU01ActNS092076Project02Year) and the Stanford Medical Scholars Research Program.

## Conflict of Interest

The authors declare that the research was conducted in the absence of any commercial or financial relationships that could be construed as a potential conflict of interest.

## Publisher's Note

All claims expressed in this article are solely those of the authors and do not necessarily represent those of their affiliated organizations, or those of the publisher, the editors and the reviewers. Any product that may be evaluated in this article, or claim that may be made by its manufacturer, is not guaranteed or endorsed by the publisher.
