# Supplementary material for: Ordinal Prediction Model of 90-Day Modified Rankin Scale in Ischemic Stroke
Source: Front Neurol. 2021 Oct 22;12:727171. doi: 10.3389/fneur.2021.727171 (PMC8569127; doi:10.3389/fneur.2021.727171)
Supplement: Supplementary file 1 [file Data_Sheet_1.docx]

**SUPPLEMENTAL FIGURES**

**Figure I: Flow diagram of study participants**

Our data was first divided into a derivation set and a validation set. We used forward selection on the up-sampled derivation set to generate an ordinal regression model. We also used five-fold cross-validation within the derivation set and used the same forward selection criteria for each fold. The goal of cross-validation was to demonstrate that the same variables remained significant even within subsets of the data. The final model was then tested on the validation set, which was not up-sampled or altered.

**Figure II: Accuracy of the 90-day mRS prediction model in five-fold internal cross-validation**

These graphs show cross-tabulations between the observed and predicted values of the 90-day mRS for the five-fold cross-validation. Four folds were used to derive the model, and one-fold was used to validate the model. Values in all graphs show **proportions**, where the columns represent the distribution of observed outcomes for a given predicted outcome. For example, in fold 1, of the patients with a predicted mRS score of 0, the observed 90-day mRS score was 0 in 44%, 1 in 38%, 2 in 19%.

**Figure III: Accuracy of the 90-day mRS prediction model in Control vs EVT**

These graphs show cross-tabulations between the observed and predicted values of the 90-day mRS for control vs endovascular therapy patients in DEFUSE 3. (A) Values show **proportions**, where the columns represent the distribution of observed outcomes for a given predicted outcome. For example, of the 12 endovascular patients with a predicted mRS score of 0, the observed 90-day mRS score was 0 in 42%, 1 in 33%, 2 in 17%, and 3 in 8%. Values in graph B show **counts** of the observed and predicted mRS scores at 90 days.

**Figure IV: 90-day mRS prediction model in patients with severe disability at discharge**

These graphs show cross-tabulations between the observed and predicted values of the 90-day mRS stratified by severe disability at discharge (NIHSS ≥ 16) in DEFUSE 3. These graphs visualize the overlap between observed and predicted scores. (A) Values in graphs show **proportions**, where the columns represent the distribution of observed outcomes for a given predicted outcome. For example, of the 16 patients with a predicted mRS score of 0, the observed 90-day mRS score was 0 in 50%, 1 in 25%, 2 in 12.5%, and 3 in 12.5%. (B) The values in the two figures in the bottom row represent counts of observed and predicted 90-day mRS scores.
